# A quantitative meta-analysis of population-based studies of premorbid intelligence and schizophrenia

**DOI:** 10.1016/j.schres.2011.06.017

**Published:** 2011-11

**Authors:** Golam M. Khandaker, Jennifer H. Barnett, Ian R. White, Peter B. Jones

**Affiliations:** a Department of Psychiatry, University of Cambridge, Cambridge, UK; b Cambridge Cognition Ltd., Cambridge, UK; c MRC Biostatistics Unit, Cambridge, UK

**Keywords:** IQ, Intelligence Quotient, SMD, Standardized Mean Difference, Schizophrenia, Intelligence, IQ, Premorbid, Child, Meta-analysis, Prospective studies, Systematic review

## Abstract

**Objective:**

A premorbid IQ deficit supports a developmental dimension to schizophrenia and its cognitive aspects that are crucial to functional outcome. Better characterisation of the association between premorbid IQ and the disorder may provide further insight into its origin and etiology. We aimed to quantify premorbid cognitive function in schizophrenia through systematic review and meta-analysis of longitudinal, population-based studies, and to characterize the risk of schizophrenia across the entire range of premorbid IQ.

**Method:**

Electronic and manual searches identified general population-based cohort or nested case–control studies that measured intelligence before onset of schizophrenic psychosis using standard psychometric tests, and that defined cases using contemporaneous ICD or DSM. Meta-analyses explored dose–response relationships between premorbid cognitive deficit (using full-scale, verbal and performance IQ) and risk of schizophrenia. Meta-regression analyses explored relationships with age of illness onset, change in premorbid intelligence over time and gender differences.

**Results:**

Meta-analysis of 4396 cases and over 745 000 controls from 12 independent studies confirmed significant decrements in premorbid IQ (effect size − 0.43) among future cases. Risk of schizophrenia operated as a consistent dose–response effect, increasing by 3.7% for every point decrease in IQ (p < 0.0001). Verbal and nonverbal measures were equally affected. Greater premorbid IQ decrement was associated with earlier illness onset (p < 0.0001). There was no evidence of a progressively increasing deficit during the premorbid period toward illness onset.

**Conclusions:**

Strong associations between premorbid IQ and risk for schizophrenia, and age of illness onset argue for a widespread neurodevelopmental contribution to schizophrenia that operates across the entire range of intellectual ability. This also suggests higher IQ may be protective in schizophrenia, perhaps by increasing active cognitive reserve.

## 1 Introduction

Identification of premorbid IQ deficits in people who will later develop schizophrenia is a key piece of evidence underpinning a developmental aspect to the disorder. This has been summarized as a neurodevelopmental theory (Murray and Lewis, 1987; Weinberger, 1987), for which there is empirical evidence (Jones et al., 1994; Weinberger, 1995). However, fundamental questions have yet to be settled. These include the absolute magnitude of premorbid IQ deficit, whether this is due to a decrement in the majority of future cases (a left-shift of this population), or whether it is due to a minority effect driven by a sub-group with conspicuously low IQ. Many studies using several designs have generally assessed mean differences between cases (or future cases) and controls (Aylward et al., 1984; Woodberry et al., 2008), rather than addressing these underlying concerns. These concerns are important because their solutions shed light on causal models of the entire syndrome of schizophrenia, and the cognitive dysfunction that is an important determinant of functional outcome.

Assessing premorbid intelligence is problematic. Previous reviews on this topic were based on studies with a number of methodological limitations which might affect both validity and generalizability of their results (Aylward et al., 1984; Woodberry et al., 2008). Case control studies are liable to selection bias, which can lead to large variations in effect size through unrepresentative comparison groups (Lubin et al., 1962; Brewer et al., 2005; Lencz et al., 2006). On the other hand, genetic high risk studies or studies of childhood psychiatric clinic attendees are often small, and may reflect atypical groups of patients; most individuals who develop schizophrenia have neither a positive family history (Gottesman and Sheilds, 1982) nor a history of psychiatric treatment in childhood. Therefore, the most accurate estimate of premorbid cognitive function in people who develop schizophrenia is likely to be gained from longitudinal population-based studies, which are more representative and less prone to selection bias than other designs.

We report a systematic review and meta-analysis of population-based studies of premorbid intelligence in schizophrenia. We aimed not only to provide an accurate estimate of the magnitude of premorbid IQ deficit, but also to characterize the variation in risk across the entire range of premorbid intellectual ability. We proposed that neurodevelopmental impairment in patients with schizophrenia operates across the normal distribution of IQ (Jones et al., 1994), and that IQ decrement would reflect this. We predicted that there would be a dose–response relationship between risk of schizophrenia and premorbid intelligence operating across the full range of IQ (as opposed to a neurodevelopmentally-impaired subgroup of patients). We proposed that the degree of premorbid IQ deficit may reflect the degree of developmental impairment at that age. Thus, we predicted that (a) the IQ deficit would be greater in individuals who show an earlier illness onset, and (b) the IQ deficit would increase with proximity between the measure of IQ and illness onset, as betrayed by an association between age and effect size once confounding by the measurement within the prodromal state was excluded. If the second scenario were true this would be consistent with a progressive IQ deficit.

## 2 Methods

### 2.1 Search strategy

Medline-PubMed, PsycINFO and Embase databases were searched to identify all studies of intelligence and schizophrenia published in English language from January 1984 (the year of the first review on this topic) (Aylward et al., 1984), until February 2011. Search terms included both MeSH terms and text words, and were: ([Premorbid OR School performance OR Childhood precursor OR Childhood antecedent] OR [IQ OR Intelligence quotient OR Intelligence tests OR Intelligence OR Cognitive OR Neuropsychological]) AND (Schizophrenia OR Psychosis). We also hand searched reference lists of included studies and related conference proceedings, and contacted prominent researchers in this field for unpublished work.

### 2.2 Study selection

Included studies (i) were general population-based cohort or nested case–control designs, (ii) measured intelligence by standard psychometric tests in childhood or before onset/diagnosis of illness, (iii) established outcome according to contemporaneous ICD or DSM, and (iv) used population based registers or cohorts to identify cases. Diagnostic outcomes were schizophrenia, schizophreniform, schizoaffective, schizotypal disorder and psychotic disorders. Studies that estimated premorbid IQ rather than directly measuring it, or that used school performance, etc. as proxies were excluded. In case of more than one published reports from the same population we included the study with longer follow up and larger sample; e.g. Zammit et al. (2004) instead of David et al. (1997) from Sweden; Urfer-Parnas et al. (2010) instead of Mortensen et al. (2005) from Denmark.

### 2.3 Data extraction

GMK examined all titles and abstracts, and obtained full texts of potentially relevant papers. GMK, JHB and PBJ read the papers and applied inclusion criteria. Data were extracted independently, and disagreements were resolved by consensus. For each report, study setting and sampling strategy, age and method of IQ measurement, follow up, attrition and method of case ascertainment were extracted.

Measures of premorbid full-scale, verbal and performance IQ in case and control groups were identified from each study. Where data were reported in a format that did not allow meta-analysis, an appropriate format was requested from the authors. When studies reported more than one measure, or repeat assessments of IQ, the most comprehensive measure was used (see Supplementary Online Methods).

### 2.4 Data synthesis

Differences in premorbid IQ between cases and controls were expressed as the standardized mean difference (SMD, Cohen's *d*), which is appropriate when studies assess the same variable but measure it in a variety of ways (Egger et al., 2001). Meta-analyses combined the SMD for premorbid full-scale, verbal and performance IQ. Heterogeneity between study samples was assessed using Cochrane's heterogeneity statistic *Q*; random effect methods were used to allow for heterogeneity between studies (Egger et al., 2001). The *I*^2^ statistic was calculated to express the fraction of variation between studies that was due to heterogeneity (Higgins et al., 2003).

To estimate the risk associated with different levels of premorbid IQ, 5 available study samples were divided into 6 groups (IQ < − 2SD, −2 to −1SD, −1 SD to 0, 0 to 1SD, 1 to 2SD and > 2SD); the 0 to 1 SD group was used as reference. Three conscript studies reported data in 9 IQ groups (Gunnell et al., 2002; Zammit et al., 2004; Tiihonen et al., 2005); we merged the groups to create 6 IQ groups reflecting similar proportions of the population as in the above six-group classification. Average IQ for each of these 6 groups was then calculated.

The dose–response relationship between IQ and risk of schizophrenia was assessed in two ways. First, we plotted the dose–response relationship in the 6 groups and combined them using multivariate random-effects meta-analysis (White, 2008). Second, logistic regression analysis was carried out to test for linearity. We fitted a quadratic model to each study, using group-specific mean IQ and its square as explanatory variables, and combined the quadratic terms in a random-effects meta-analysis: a mean significantly different from zero indicates overall non-linearity, while significant heterogeneity indicates that non-linearity occurs in at least some studies but differs between studies.

Longitudinal data with repeat measures of IQ on the same sample were available from only three studies (Jones et al., 1994; Crow et al., 1995; Cannon et al., 2002). In order to assess any change in IQ during the premorbid period, we plotted the Z scores at different age points to examine any change in IQ deficit over time. In addition, we separately meta-analyzed studies where IQ testing occurred ‘early’ (between age 7 and 12 years) versus ‘late’ (16–19 years), and tested the difference between these effect sizes (Altman and Bland, 2003). Meta-regression was also used to assess whether the magnitude of deficit in premorbid IQ correlated with the age of IQ testing.

Meta-regression was used to examine the association between degree of IQ deficit and age of onset of schizophrenia. To test whether premorbid IQ decrement differed between men and women we carried out meta-regression of SMD on proportion of men among cases, adjusting for the age of IQ testing. Publication bias was assessed using funnel plots and Egger's test (Egger et al., 1997). Data were analyzed using Comprehensive Meta-Analysis Version 2.0 (Biostat Inc, Englewood, NJ, USA) and STATA 10 (StataCorp LP, Texas, USA).

## 3 Results

Electronic search identified 471 studies. On the basis of title and abstracts 25 (5.3%) potentially eligible studies were identified, of which 13 met inclusion criteria. One additional study was obtained from correspondence with authors. In total 14 studies were included in the review (Table 1).

### 3.1 Premorbid IQ in schizophrenia

**Fig. 1:**
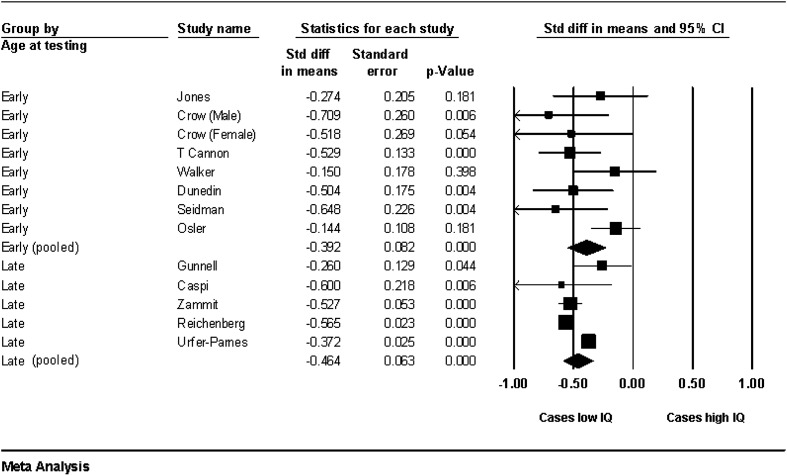
Forest plot of premorbid full-scale IQ among a total of 4396 cases and 745 720 controls. Note: Random effect meta-analysis. Size of the squares is proportional to the weight of study. The diamonds indicate pooled SMD from two groups of studies defined by age of IQ testing, as ‘early’—7 to 12 years and ‘late’—16 to 19 years. The vertical line on x-axis at 0.00 indicates the point of no difference in premorbid IQ between schizophrenia cases and controls.

A measure of premorbid full-scale IQ was available from 12 studies, which included 4396 cases and 745 720 controls (Jones et al., 1994; Crow et al., 1995; Cannon et al., 2000; Gunnell et al., 2002; Walker et al., 2002; Caspi et al., 2003; Zammit et al., 2004; Reichenberg et al., 2005; Seidman et al., 2006; Osler et al., 2007; Urfer-Parnas et al., 2010). Standardized Mean Difference (SMD) in premorbid full-scale IQ between cases and controls was − 0.43 (95% confidence interval (CI) −0.53 to − 0.34), p < 0.0001; see Fig. 1. This equates to a mean premorbid IQ of 93.6 in people who develop schizophrenia, compared with a population mean of 100 (SD 15). There was however, significant heterogeneity in effect size between studies, (p < 0.001; *I*^*2*^ = 77%).

### 3.2 Verbal and performance IQ

Both verbal and performance IQ were significantly lower in individuals who later developed schizophrenia. Ten studies reported valid measures of both premorbid verbal and performance IQ (Jones et al., 1994; Crow et al., 1995; Cannon et al., 2002; Gunnell et al., 2002; Caspi et al., 2003; Niendam et al., 2003; Zammit et al., 2004; Reichenberg et al., 2005; Seidman et al., 2006; Osler et al., 2007). Meta-analysis of verbal IQ included 2349 cases and 669 096 controls and showed a SMD of − 0.43 (95% CI − 0.52 to − 0.33), p < 0.0001. Meta-analysis of performance IQ included 2477 cases and 691 965 controls and showed a SMD of − 0.43 (95% CI − 0.55 to − 0.31), p < 0.0001. There was no significant difference between deficits in premorbid verbal and performance IQ (p = 0.46). Heterogeneity of effects among studies of verbal IQ was marginally non-significant (p = 0.08; *I*^*2*^ = 41%), but significant heterogeneity was observed among studies of performance IQ (p = 0.001; *I*^*2*^ = 65%).

### 3.3 Dose response relationship between IQ and risk of schizophrenia

**Fig. 2:**
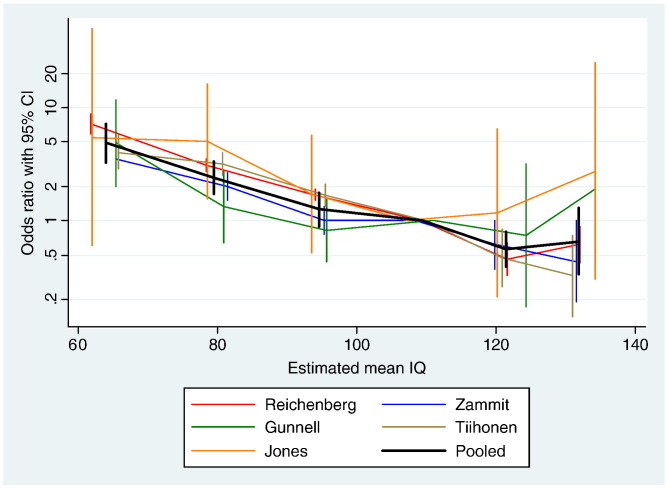
Odds ratio for risk of schizophrenia across six IQ categories. Note: Estimated mean IQ refers to mean IQ of each category calculated according to proportion of people in the category. IQ category 0–1SD is used as reference.

Data from five cohorts were available for this analysis (Jones et al., 1994; Gunnell et al., 2002; Zammit et al., 2004; Reichenberg et al., 2005; Tiihonen et al., 2005). Compared with the group with IQ between the population mean and one SD above the mean (i.e. IQ 100 to 115), the risk of developing schizophrenia was more than doubled among individuals with IQ between 70 and 85 (Odds ratio (OR) 2.36, 95% CI 1.59 to 3.49), while risk was nearly five-fold for those with IQ below 70 (OR 4.78, 95% CI 3.19 to 7.13); see Fig. 2. High premorbid intelligence reduced the risk of schizophrenia; OR for those with IQ between 115 and 130 was 0.55 (95% CI 0.38 to 0.81), and those with IQ over 130 was 0.61 (95% CI 0.29 to 1.29).

Logistic regression of five studies assuming a common slope showed a 3.7% increase in risk of schizophrenia with each one-point decrease in IQ (95% CI 3.4% to 3.9%, p < 0.0001). Examination of whether a non-linear relationship between IQ score and schizophrenia provided a better fit for the data was made by inclusion of a quadratic term in the logistic regression model. Quadratic effects were significantly heterogeneous between studies (p = 0.002). Although the overall quadratic effect was not significant, study-specific quadratic effects for two studies were significantly positive, indicating a weaker relationship between IQ and schizophrenia at higher IQs (quadratic coefficient 0.001, p < 0.001) (Gunnell et al., 2002); (quadratic coefficient 0.0001, p = 0.04) (Reichenberg et al., 2005); while one study suggested a possibly stronger relationship between IQ and schizophrenia at higher IQs (quadratic coefficient − 0.0002, p = 0.06) (Tiihonen et al., 2005).

### 3.4 Age of onset of schizophrenia

**Fig. 3:**
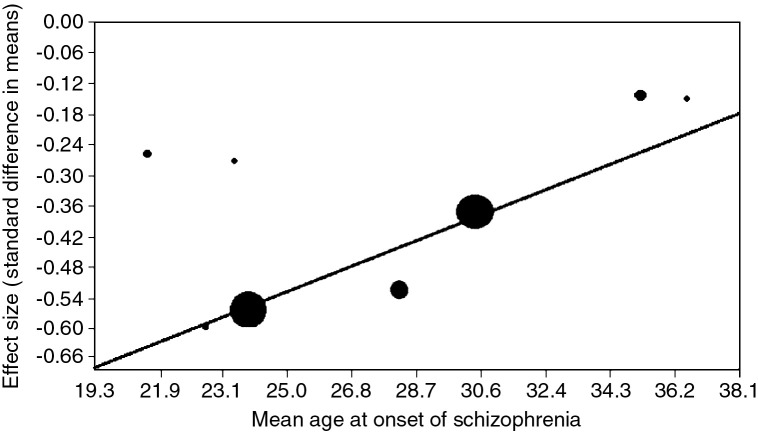
Meta-regression of age of onset of schizophrenia on standardized mean difference in premorbid full-scale IQ. Note: Each circle represents a study. Circle size is proportional to study weight.

Both full-scale IQ and age of illness onset were available from 8 studies (Jones et al., 1994; Gunnell et al., 2002; Walker et al., 2002; Caspi et al., 2003; Zammit et al., 2004; Reichenberg et al., 2005; Osler et al., 2007; Urfer-Parnas et al., 2010). Meta-regression showed strong evidence for greater case–control difference in samples with an earlier mean age of onset (slope = 0.03, SE 0.004, z = 5.92, p < 0.0001; see Fig. 3).

### 3.5 Is the IQ deficit progressive?

To assess change in IQ during the premorbid period we looked at both within and between study changes in IQ over time. Repeated measures of IQ within the same individuals were available from three cohorts totalling 86 cases. IQ was tested at age 8, 11 and 15 years in the 1946 British birth cohort (Jones et al., 1994); at 5, 7, 11, and 13 in the Dunedin birth cohort (Cannon et al., 2002); and at 8, 11 and 16 in the 1958 British birth cohort (Crow et al., 1995). SMD in full-scale IQ was plotted at these time points, however, there was no evidence of significant change in IQ over time.

IQ was measured earlier in the prospective birth cohort studies (between age 7 and 12) than the conscript cohorts (between 16 and 19 years). We carried out separate meta-analysis dividing the samples into two groups according to age of IQ testing (early versus late). There was no significant difference in premorbid IQ deficit between two groups (z = 1.31, p = 0.10). Meta-regression of SMD in full-scale IQ on age at the time of IQ testing (years) found no evidence for a greater IQ deficit with advancing age (slope = 0.007, SE 0.006, z = 1.13, p = 0.26).

### 3.6 Gender effects on premorbid IQ

The review by Aylward et al*.* reported a higher deficit in premorbid IQ among male cases of schizophrenia (Aylward et al., 1984). We carried out a meta-regression of SMD in full-scale IQ on proportion of men in cases based on 12 samples, adjusting for the age of IQ testing (Jones et al., 1994; Crow et al., 1995; Cannon et al., 2000; Gunnell et al., 2002; Walker et al., 2002; Caspi et al., 2003; Zammit et al., 2004; Reichenberg et al., 2005; Seidman et al., 2006; Osler et al., 2007; Urfer-Parnas et al., 2010). We found no evidence of significant difference in premorbid IQ between studies with different proportions of men (slope = 0.0016, SE 0.0025, p = 0.55). There was also no evidence of association between mean age of onset of a sample and the male:female ratio.

### 3.7 Evaluation of publication bias

Inspection of a funnel plot and formal assessment using Egger's test found no significant indication of publication bias among reports of full-scale IQ (Egger's intercept 0.98, p = 0.08), verbal IQ (p = 0.84) or performance IQ (p = 0.06).

## 4 Discussion

The neurodevelopmental hypothesis of schizophrenia argues that aberrant brain development is detectable in subtle cognitive, motor and other impairments present early in life, and that these differences are markers of the neural process that predisposes or leads to psychotic symptoms following further normal and/or abnormal developmental events (Murray and Lewis, 1987; Weinberger, 1987). The present meta-analysis of robust population-based datasets, involving 4396 cases and 745 720 controls confirmed the presence of an IQ deficit of around 0.4 standard deviations among young people who will later develop schizophrenia. This is a large effect at the population level. Moreover, we observed a linear association between schizophrenia and IQ, equating to a 3.7% increase in risk for every 1-point decrease in premorbid IQ. These findings cannot be explained only by a severely impaired subgroup, and are consistent with previous reports of a left-shift of the entire distribution of premorbid IQ score in schizophrenia (Jones et al., 1994; David et al., 1997).

Unlike many putative risk factors for schizophrenia, this meta-analysis showed strikingly consistent associations between premorbid IQ and schizophrenia (Fig. 1). Nonetheless, some heterogeneity in effect size was present. Possible sources of heterogeneity include differences in measures of IQ and testing procedures, sample types (cohort versus conscript studies), and length of follow up. For example, studies that used school-based group IQ tests reported a smaller decrement (Walker et al., 2002; Osler et al., 2007) than studies that used individual assessment, such as WISC, at a similar age (Cannon et al., 2000, 2002). Nonetheless, the remarkably similar effect size obtained from aggregation of verbal and nonverbal IQ measures suggests that differences in testing method may be relatively unimportant.

Greater premorbid IQ deficits were strongly associated with an earlier average age of illness onset (Fig. 3). We acknowledge that mean age of onset in longitudinal studies can be influenced by length of follow up. However, our finding is consistent with previous case control studies (Pollack, 1960; Offord and Cross, 1971), and the only previous review that examined this association (Aylward et al., 1984). One conscript study reported only a modest deficit in premorbid IQ in cases of early onset schizophrenia (Gunnell et al., 2002), which can be explained by its relatively short duration of follow. Moreover, most military conscript cohorts excluded individuals presenting with mental illness, mental retardation, epilepsy or any severe disability at the time of draft assessment (Gunnell et al., 2002; Zammit et al., 2004; Reichenberg et al., 2005; Urfer-Parnas et al., 2010). This may have biased the samples away from the early onset cases of schizophrenia, which may be the most cognitively impaired, and hence underestimated the association between low IQ and schizophrenia. Nonetheless, we found two approximately linear relationships, between premorbid IQ and risk for schizophrenia, and between premorbid IQ and age of onset of schizophrenia.

These findings point toward a widespread neurodevelopmental contribution to schizophrenia that operates across the entire range of intellectual ability. This is in contrast to previous suggestions that neurodevelopmental abnormalities may be confined to (Murray et al., 1992), or more prominent (Dean et al., 2003), in a subgroup of schizophrenia cases characterized by poor premorbid intelligence, early illness onset and male predominance. This is not to suggest that other factors, alone or in combinations, are not important causes of schizophrenia; and that discrete combination of genetic and environmental factors may be involved. However, the data are consistent with a continuity of developmental risk rather than a sub-type. The majority of causal constellations are either manifest as, or mediated by a cognitive or IQ-related factor. Such an effect may involve either risk or protection, as discussed, below.

IQ in military conscripts was tested later (16–19 years) than in birth cohorts (6–12 years). To eliminate the possibility of reverse causality (i.e. low IQ as a result of early disease process or prodromal state) three conscript studies excluded those who developed psychosis within one (Reichenberg et al., 2005; Urfer-Parnas et al., 2010), or two (Caspi et al., 2003) years after cognitive assessment. However, the possibility of some prodromal cases in the remaining two conscript studies cannot be ruled out (Gunnell et al., 2002; Zammit et al., 2004). The mean effect size in conscript studies appears to be slightly larger than that of birth cohorts (− 0.46 versus −0.39), although this difference was not statistically significant. As the mean effect size of IQ deficit in first-episode schizophrenia is − 0.89 (Rajji et al., 2009), one could argue that our findings suggest a subtle but gradual deterioration of intellectual function over the premorbid period. Progressive deterioration in premorbid IQ is in keeping with both early static and dynamic brain lesion according to the neurodevelopmental hypothesis of schizophrenia. A static lesion can interact with other environmental factors (as well as normal maturational processes) to result in deterioration in intellectual performance during disease prodrome. Alternatively in a ‘dynamic model’ developmental mechanisms such as apoptosis and neuronal pruning can give rise to cognitive deterioration much earlier in the premorbid period (Woods, 1998). However, this hypothesis can be tested conclusively only by repeated assessment within the same individuals. Such longitudinal data were available from only three cohorts (totalling 86 cases), so further studies with larger samples are required. Our own view tends toward a more dynamic model involving a developmental view of neural connectivity (Ridler et al., 2006) that is discussed, below.

Additional possible sources of heterogeneity between studies include differences in case definition and ascertainment, and gender distribution between samples. Studies that used a narrower definition of schizophrenia, or ascertained cases through hospitalization, may detect a subset of more severe cases, resulting in larger effect size (Crow et al., 1995; Seidman et al., 2006). Conversely, the use of a broader diagnostic outcome (ICD 10 code F20–29) may have diluted the effect size in one study (Walker et al., 2002). Despite sex differences in cognitive development (Belenky et al., 1986) and age of risk for schizophrenia (Kirkbride et al., 2006), we found no significant association between gender ratio and effect size. However, these meta-regression analyses lacked power due to limited data-sets. In contrast to a previous review (Aylward et al., 1984), we found no difference in the magnitude of verbal versus nonverbal cognitive decrements, although estimates of verbal IQ showed less heterogeneity between samples than those of nonverbal IQ, perhaps due to differences in the range of tests used to estimate these scores. A limitation of this review is lack of individual participant data, which did not allow us to account for confounding factors in the meta-analysis and meta-regression analyses (Thompson and Higgins, 2005). However, all individual studies adjusted their results for several confounding factors (Table 1), and all reported an effect in the same direction (Fig. 1).

An alternative explanatory model is that IQ is a protective factor, buffering the impact of separate processes that, themselves, modify (increase) the risk for psychosis. Such an interactive model draws upon theories of cognitive reserve, a concept proposed to account for apparent disparity between the degree of brain pathology and its clinical manifestations (Stern, 2002). For example, up to 25% of elderly people who perform normally in neuropsychological tests prior to death meet criteria for Alzheimer's disease in post-mortem histopathological examinations (Ince, 2001), suggesting that this degree of pathology does not invariably result in clinical dementia. In neuropsychiatric disorders such as schizophrenia cognitive reserve may be associated with the risk of developing illness, as well as symptom manifestation and long term functional outcome (Barnett et al., 2006). While the passive model of cognitive reserve concerns individual differences in brain structure (e.g. brain volume or neuron density), the active model considers differences in the functionality of brain processing. Active cognitive reserve can be measured by performance in intelligence tests, educational and occupational attainment, etc. We found that risk of schizophrenia was not restricted to the lower end of the intelligence spectrum. Rather, we observed strong evidence for a linear association, whereby those with average IQ were at significantly increased risk of developing schizophrenia than those with higher IQ (Fig. 2). This suggests cognitive reserve may be an active process in schizophrenia, and its protective effects may operate throughout the range of intelligence spectrum.

Our results certainly support the view that schizophrenia includes an element of abnormal neurodevelopment with phenotypic expression starting very early in life (Murray and Lewis, 1987; Weinberger, 1987; Jones et al., 1994). The existence of two approximately linear relationships, where lower premorbid IQ is associated with both increased risk and earlier onset of schizophrenia, suggests a gradient of neurodevelopmental impairment where the relationship between position on the distribution of cognitive ability and frequency of schizophrenia is similar to that between, e.g., blood pressure and stroke (Jones et al., 1994). These findings fit the model of psychosis as a continuum (van Os et al., 2009), where the association between IQ and psychotic-like experiences extends beyond schizophrenia. The same spectrum of neurodevelopmental impairment that is seen in schizophrenia may also underlie results from general population studies, where childhood IQ is also associated with risk for adult (Kremen et al., 1998) and childhood (Horwood et al., 2008) non-clinical psychotic symptoms. Neuroimaging studies based on a Finnish birth cohort showed that developmental failure to establish anatomical connectivity in premotor cortex, and abnormal integration of fronto-cerebellar-thalamic circuits may underlie both delayed infant motor development and adult cognitive dysfunction in schizophrenia (Ridler et al., 2006), suggesting that lower IQ in this context, before and after the onset of disorder, is a marker of abnormal connectivity implicated in the disorder. Thus, lower premorbid IQ might reflect the underlying neurodevelopmental abnormality, which in some individuals leads to the development of psychotic symptoms, alone or in combination of other genetic and environmental factors.

On the whole, the studies reviewed here represent a major advance in methodology of studies of schizophrenia epidemiology. However, future studies on this topic should also include repeat measures of IQ on same individuals using adequate samples; address the question as to whether the deficit is a general one involving the concept of ‘G’ or whether specific sub-function(s) of cognition are uniquely involved. Furthermore, the determinants of the IQ distribution and normal neurodevelopment may conceal important aspects of the genesis of later neuropsychiatric disorders such as schizophrenia. The triangulation of cognitive epidemiological data with neuroimaging and other neuroscience disciplines would help to elucidate underlying such pathological processes.

The following are the supplementary materials related to this article.

Data extraction Niendam et al. (2003) reported individual subtest scores of the Wechsler Intelligence Scale for Children (WISC) for a subgroup of a larger cohort. Cannon et al. (2000) previously reported only the full scale IQ for that cohort. We used the larger sample in full-scale IQ analysis, and selected the Vocabulary and Block Design subtests for verbal and performance IQ analyses, respectively. Cannon et al. (2002) reported a 26 year follow up of the Dunedin birth cohort which included 33 cases of schizophreniform disorder. We used premorbid verbal and performance IQ measures (WISC-R) at age 7 years from that study. However, in the analysis of full scale IQ we used 32 year follow up data, which included full scale IQ (WISC-R) averaged across testing ages 7, 9, 11 and 13 years, in 35 cases of schizophreniform disorder and 583 healthy controls (personal communication; Prof. T. Moffitt).

## Role of funding source

Golam Khandaker is supported by a grant from the Wellcome Trust (Clinical PhD Programme, grant number 094790/Z/10/Z). Peter Jones is also supported by the Wellcome Trust (095844/Z/11/Z & 088869/Z/09/Z) and NIHR (RP-PG-0606-1335). The funding organizations had no further role in study design; in the collection, analysis and interpretation of data; in the writing of the report; and in the decision to submit the paper for publication.

## Contributors

Golam Khandaker carried out literature search, analysis and wrote the first draft of the manuscript. Jenny Barnett and Ian White contributed to the analysis and revision of manuscript. Peter Jones conceived the study, contributed to manuscript and provided overall supervision for the project. All authors contributed to and have approved the final manuscript.

## Declaration of interest

Golam Khandaker and Ian White report no competing interest. Jenny Barnett is an employee of Cambridge Cognition Ltd. Jenny Barnett and Peter Jones are co-inventors on patent PCT/GB2005/003279 (methods for assessing psychotic disorders). Peter Jones has received research support from GlaxoSmithKline, and directs the National Institute for Health Research Collaborations for Leadership in Applied Health Research and Care for Cambridgeshire and Peterborough.

## Figures and Tables

**Table 1:** Studies included in the systematic review of premorbid intelligence and Schizophrenia.

Study	Design and setting	Diagnostic criteria and outcome	Case (n)	Control (n)	% of cases male	Age at follow up (years)	IQ test age (years)	Mean age of onset, years (SD)	IQ tests	Case identification	Adjustment for confounding
Jones et al. (1994)	PC^a^—British 1946 birth cohort	DSM III R schizophrenia	24	4133	66.6	43	8, 11, 15	23.4 (2.7)	1st principal component^b^	Questionnaire, interview, hospital discharge register, present state examination	Age, social class
Crow et al. (1995)	PC—British 1958 birth cohort	PSE CATEGO^c^ schizophrenia	29	1446	51.7	28	7, 11, 16	–	General ability test	Hospital discharge register	Social class
Cannon et al. (2000)	PC—NCPP: Philadelphia cohort, USA^d^	DSM IV Schizophrenia or schizoaffective	57	5829	72	19–36	4, 7	–	Wechsler intelligence scale for children (WISC)	Hospital discharge register	Socio-demographic, obstetric, behavioral factors
Walker et al. (2002)	RC^e^—Scottish mental ability survey 1932	ICD10: F20–29 Schizophrenia and related conditions	32	3764	65.5	66	11	36.5 (16.6)^f^	Moray house test	Hospital discharge register	Age
Cannon et al. (2002)	PC—Dunedin birth cohort, New Zealand	DSM IV Schizophreniform	33	598	–	32	3, 5, 7, 9, 11	–	Revised Wechsler intelligence scale for children (WISC-R)	Diagnostic interview schedule (DIS)	Socioeconomic, obstetric and maternal factors
Gunnell et al. (2002)	RC—Swedish conscript cohort	ICD 10 schizophrenia	60	109 491	100	18–25	18	20.8 (1.5)^f^	Synonym and visuospatial test	National Inpatient Discharge Register	Age, obstetric and maternal factors, parental education
Caspi et al. (2003)	NCC^g^—Israeli conscript cohort	DSM IV schizophrenia	44	44	75	19–31	16–17	22.5 (3.5)^f^	Modified Raven's progressive matrices, Otis type verbal IQ test	Structured Clinical Interview for DSM (SCID)	Sex, country of origin, age, education
Niendam et al. (2003)^h^	NCC—NCPP: Philadelphia cohort, USA	DSM IV schizophrenia or schizoaffective	32	201	34	19–36	7	–	Vocabulary and block design	Hospital discharge register	Age, socio-economic status
Zammit et al. (2004)	RC—Swedish conscript cohort	ICD 8 and ICD 9 Schizophrenia	362	49 161	100	45–47	18	28.2 (5.9)^f^	Visuospatial test	National Inpatient discharge register	–
Reichenberg et al. (2005)	RC—Israeli conscript cohort	ICD 10 Schizophrenia	1856	549 466	76	24–34	16–17	23.8 (3.7)^f^	Verbal and non-verbal analogies	National Inpatient discharge register	Sex, socioeconomic status
Tiihonen et al. (2005)^i^	RC—Finnish conscript cohort	ICD 8 and ICD 9 schizophrenia	607	189 027	100	27	19.9	–	Verbal, arithmetic and visuospatial reasoning tests	National inpatient discharge register	Age, over-all test performance
Seidman et al. (2006)	NCC—NCPP: New England cohort, USA	DSM IV schizophrenia	31	61	79.4	28	7	–	Wechsler intelligence scale for children (WISC)	DIS, SCID, hospital discharge register	Sex, ethnicity, socioeconomic status
Osler et al. (2007)	PC—Danish longitudinal study	ICD 10 schizophrenia	87	6790	100	19–49	12	35.2 (9.6)^f^	Härnquist test	National inpatient discharge register	Birth weight, social class, education, marital status, employment
Urfer-Parnas et al. (2010)	NCC—Danish conscript cohort	ICD 8 to ICD 10 schizophrenia	1779	20 531	100	43–54	19.5	30.4 (7.8)^f^	Børge Priens Prøve (letter matrices, verbal analogies, number series, geometric figures)	National inpatient discharge register	–

a PC—Prospective cohort study.

b 1st principal component derived from all tests used at each age which included tests for verbal, nonverbal and mathematical skills.

c Present state examination and CATEGO.

d National Collaborative Perinatal Project (NCPP)—USA.

e RC—Retrospective cohort study.

f Age at the time of first hospital admission for schizophrenia.

g NCC—Nested case–control study.

h Study sample is a sub-set of T Cannon et al. (2000) used in the meta-analysis of premorbid verbal and performance IQ.

i Study used only in the dose–response analysis as data was not available in appropriate format to use in other analyses.
