# Supplementary material for: A quantitative meta-analysis of population-based studies of premorbid intelligence and schizophrenia
Source: Schizophr Res. 2011 Nov;132(2-3):220–7. doi: 10.1016/j.schres.2011.06.017 (PMC3485562; doi:10.1016/j.schres.2011.06.017)
Supplement: Data extraction — Niendam et al. (2003) reported individual subtest scores of the Wechsler Intelligence Scale for Children (WISC) for a subgroup of a larger cohort. Cannon et al. (2000) previously reported only the full scale IQ for that cohort. We used the larger sample in full-scale IQ analysis, and selected the Vocabulary and Block Design subtests for verbal and performance IQ analyses, respectively. Cannon et al. (2002) reported a 26 year follow up of the Dunedin birth cohort which included 33 cases of schizophreniform disorder. We used premorbid verbal and performance IQ measures (WISC-R) at age 7 years from that study. However, in the analysis of full scale IQ we used 32 year follow up data, which included full scale IQ (WISC-R) averaged across testing ages 7, 9, 11 and 13 years, in 35 cases of schizophreniform disorder and 583 healthy controls (personal communication; Prof. T. Moffitt). [file mmc1.doc]

**Methods**

**Data Extraction**

Niendam *et al* (2003) reported individual subtest scores of the Wechsler Intelligence Scale for Children (WISC) for a subgroup of a larger cohort. Cannon, T. *et al* (2000) previously reported only the full scale IQ for that cohort. We used the larger sample in full-scale IQ analysis, and selected the Vocabulary and Block Design subtests for verbal and performance IQ analyses, respectively. Cannon, M. *et al* (2002) reported a 26 year follow up of the Dunedin birth cohort which included 33 cases of schizophreniform disorder (18). We used premorbid verbal and performance IQ measures (WISC-R) at age 7 years from that study. However, in the analysis of full scale IQ we used 32 year follow up data, which included full scale IQ (WISC-R) averaged across testing ages 7, 9, 11 and 13 years, in 35 cases of schizophreniform disorder and 583 healthy controls (personal communication; Prof. T. Moffitt).
